# Emergence and Spread of Enterobacterales with Multiple Carbapenemases after COVID-19 Pandemic

**DOI:** 10.3390/pathogens12050677

**Published:** 2023-05-03

**Authors:** Branka Bedenić, Josefa Luxner, Haris Car, Sanda Sardelić, Maja Bogdan, Dijana Varda-Brkić, Sandra Šuto, Andrea Grisold, Nataša Beader, Gernot Zarfel

**Affiliations:** 1 Department of Microbiology, University of Zagreb School of Medicine, 10000 Zagreb, Croatia; 2 Clinical Department for Clinical and Molecular Microbiology, University Hospital Center Zagreb, 10000 Zagreb, Croatia; 3 Institute for Hygiene, Microbiology and Environmental Medicine, Medical University Graz, 8010 Graz, Austria; 4 Zagreb Health School, 10000 Zagreb, Croatia; 5 Department of Microbiology, University Hospital Centre Split, 21000 Split, Croatia; 6 Department of Microbiology, University Hospital Centre Osijek, 31000 Osijek, Croatia; 7 Department of Microbiology, Andrija Štampar Public Health Institute, 10000 Zagreb, Croatia

**Keywords:** Enterobacterales, multiple carbapenemases, resistance, OXA-48, NDM, VIM

## Abstract

Resistance to carbapenems in *Enterobacterales* has become a matter of the highest concern in the last decade. Recently, *Enterobacterales* harboring multiple carbapenemases were detected in three hospital centers in Croatia and in the outpatient setting, posing a serious therapeutic challenge for clinicians. In this study, we analyzed eight *Klebsiella pneumoniae* and two *Enterobacter cloacae* complex isolates with multiple carbapenemases, with regard to antibiotic susceptibility, β-lactamase production and plasmid content. The isolates demonstrated uniform resistance to amoxicillin/clavulanate, piperacillin/tazobactam, cefuroxime, ceftazidime, cefotaxime, ceftriaxone and ertapenem. Among novel β-lactam/inhibitor combinations, ceftazidime/avibactam exhibited moderate activity, with 50% of isolates susceptible. All isolates demonstrated resistance to imipenem/cilastatin/relebactam, and all but one to ceftolozane/tazobactam. Four isolates exhibited a multidrug-resistant phenotype (MDR), whereas six were allocated to an extensively drug-resistant phenotype (XDR). OKNV detected three combinations of carbapenemases: OXA-48+NDM (five isolates), OXA-48+VIM (three isolates) and OXA-48+KPC (two isolates). Inter-array testing identified a wide variety of resistance genes for β-lactam antibiotics: *bla*_CTX-M-15_, *bla*_TEM_, *bla*_SHV_, *bla*_OXA-1_, *bla*_OXA-2_, *bla*_OXA-9_, aminoglycosides: *aac*6, *aad*, *rmt*, *arm* and *aph*, fluoroquinolones: *qnr*A, *qnr*B and *qnr*S, sulphonamides: *sul*1 and *sul*2 and trimethoprim: *dfrA5, dfrA7, dfrA14, dfrA17 and dfrA19*. *mcr* genes were reported for the first time in Croatia. This study demonstrated the ability of *K. pneumoniae* and *E. cloacae* to acquire various resistance determinants under the selection pressure of antibiotics widely used during the COVID-19 pandemic. The novel inter-array method showed good correlation with OKNV and PCR, although some discrepancies were found.

## 1. Introduction

Resistance to carbapenems in Enterobacterales has become a matter of the highest concern in the last decade [1]. It has mainly been associated with the production of carbapenemases belonging to Ambler class A serine β-lactamases (KPC, GES, SME, IMI, NMC), class B metallo-β-lactamases (MBL) of the IMP, VIM or NDM family or OXA-48-like β-lactamases belonging to the class D [1]. Genes encoding carbapenemases can be located either on the chromosome or on plasmids with different replicon types (IncFII, Inc/L/M, IncP, IncN and IncA/C) [2]. The plasmids encoding carbapenemases often also carry the *bla*_ampC_, *bla*_ESBL_ and *qnr* genes and genes encoding acetylases, adenylases and phosphorylases, which hydrolyze aminoglycosides and render them inactive [1].

Since 2011, the emergence and rapid spread of carbapenemase-producing Enterobacterales was reported in Croatia, first with NDM-1 [3], KPC-2 [4,5] and VIM-1 in the early stage of dissemination [6,7], and rapid expansion of OXA-48 in the recent period [8,9].

Recently, *Enterobacterales* harboring multiple carbapenemases, usually associated with the extensively resistant phenotype (XDR), were detected in three hospital centers in Croatia and in the outpatient setting, posing a serious therapeutic challenge for clinicians. The strains with double carbapenemases occurred only rarely before 2020, but the number of the strains increased during and after the COVID-19 pandemic due to increased antibiotic usage. There were a lot of mechanically ventilated patients in hospitals with secondary bacterial infections receiving high dosages of carbapenems and colistin, which exerted selection pressure favoring the acquisition of carbapenemase-encoding genes.

The aim of the present study was to analyze eight *Klebsiella pneumoniae* and two *Enterobacter cloacae* complex isolates with multiple carbapenemases through conventional PCR and sequencing, followed by an evaluation of the new inter-array test- system.

## 2. Material and Methods

### 2.1. Bacterial Isolates

Ten bacterial isolates were collected in four centers: University Hospital Centre Zagreb (UHCZ), Andrija Štampar Public Health Institute, University Hospital Centre Split (UHCS), located in the southern region of Croatia, and University Hospital Centre Osijek (UHCO) in the eastern region of Croatia. They originated from various clinical specimens including clinically relevant (urine, blood cultures, wound swab) and colonization specimens (throat swab) during the period 2014 to 2022. Two isolates were recovered prior to COVID-19 and eight during the pandemic. All isolates from the affected medical facilities confirmed to possess a combination of carbapenemases through immunochromatographic OKNV (OXA-48, KPC, NDM, VIM) testing [10] were sent to the UHCZ for microbiological and molecular analysis. The isolates were identified through MALDI-TOF mass spectrometry (matrix-assisted laser desorption ionization–time-of-flight mass spectrometry, Bruker, Illinois, USA). The inter-array chip test and the MLST were carried out at the Institute for Hygiene, Microbiology and Environmental Medicine of the University in Graz. The study was approved by the ethical committee of the University Hospital Centre Zagreb (class: 8.1-15/122-2, number: 02/21 AG). All microbiological samples were taken as part of standard routine diagnosis. No written informed consent was necessary for this type of study.

### 2.2. Antimicrobial Susceptibility Testing and Phenotypic Tests for Detection of ESBLs, Plasmid-Mediated AmpC β-Lactamases and Carbapenemases

All isolates were tested first using the disk diffusion test according to EUCAST in the context of routine diagnostic, and later, for research purposes, broth dilution was carried out for amoxicillin/clavulanate, piperacillin/tazobactam, cefuroxime, ceftazidime, cefotaxime, ceftriaxone, cefepime, imipenem, meropenem, gentamicin, ciprofloxacin and colistin.

The minimum inhibitory concentrations (MICs) of the agents listed in Table 1 were evaluated using the broth microdilution method with the inoculum size of 5 × 10^5^ CFU/mL according to CLSI standards [11] for all antibiotics except colistin, for which the EUCAST standard was applied [12]. The susceptibility to trimethoprim/sulfamethoxazole, cefoxitin, aztreonam, ertapenem, amikacin, ceftazidime/avibactam, ceftolozane/tazobactam and imipenem/cilastatin/relebactam was determined using the disk diffusion method. Additionally, the E-test was applied for cefiderocol, ceftazidime/avibactam and ceftolozane/tazobactam. The strains were classified as multidrug-resistant (MDR), extensively drug resistant (XDR) or pan-drug resistant (PDR) [13].

A double disk synergy test (DDST) [14] and CLSI combined disk test with the addition of clavulanic acid were performed to detect extended-spectrum β-lactamases (ESBLs) [11]. Overproduction of chromosomal or production of plasmid-mediated AmpC-β-lactamases were detected through the combined disk test using cephalosporin disks combined with PBA (3-aminophenylboronic acid [15]. A modified Hodge test (MHT) was used to screen for the production of carbapenemases [16]. Enhanced growth was interpreted as positive for carbapenemase production, while no enhancement was negative for carbapenemase. The presence of cloverleaf-shaped indentation was considered a positive MHT. The isolates were additionally phenotypically screened for a specific class of carbapenemases by performing combined disk testing using four disks of meropenem, one without and the other three with 3-aminophenylboronic acid test (PBA), 0.1 M EDTA or both EDTA and PBA to screen for KPC, MBLs, or the simultaneous production of KPC and MBLs, respectively [17].

### 2.3. Genotyping by Inter-Array Kit CarbaResist

The isolates were subjected to genotyping using an inter-array chip according to the manufacturer’s recommendations (Inter-array fzmb GmbH, Bad Langensalza, Germany). The inter-array genotyping kit CarbaResist allows the DNA-based detection of the most common β-lactamases and other resistance genes of multidrug-resistant Gram-negative bacteria from bacterial cultures. After the isolation of RNA-free, unfragmented genomic DNA from pure material, the DNA was amplified and internally labelled with biotin-dUDP using the linear PCR amplification protocol and only the antisense primer of the different targets. The results were single-stranded DNA (ssDNA) reaction products. In the next step, this biotin-labelled ssDNA was transferred into an ArrayWell and hybridized to DNA oligonucleotide microarrays with 230 probes for different carbapenemases, *bla*_ESBL_ and *bla*_ampC_ genes, as well as other relevant antibiotic resistance genes. After hybridization and subsequent washing, HRP conjugated streptavidin bound to the hybridized biotin-labelled ssDNA strains and visualized them in a subsequent enzymatic reaction. The evaluation of the spots and their intensities was achieved automatically on the basis of a digital image of the microarray with an INTER-VISION Reader. The overall samples were automatically analyzed for the presence or absence of specific probes, cross-checked against a database and then information on existing resistances and possible bacterial species was outputted.

### 2.4. Molecular Detection of Resistance Genes

Template DNA was extracted through the boiling method. The genes encoding resistance to β-lactams including broad-spectrum and extended-spectrum β-lactamases (*bla*_SHV_, *bla*_TEM_, *bla*_CTX-M_) [18,19,20,21], plasmid-mediated AmpC- β-lactamases [22], class A (*bla*_KPC_,), class B (*bla*_VIM,_ *bla*_IMP_ and *bla*_NDM_) and class D carbapenemases or carbapenem-hydrolyzing oxacillinases (*bla*_OXA-48-like_) [23] and to fluoroquinolones (*qnr*A, *qnr*B, *qnr*S) [24] were amplified and sequenced as previously reported. One negative SHV-PCR strain of *K. pneumoniae* was additionally tested with the additional primers SE5 and SB35 for the amplification of *bla*_SHV_ genes as described previously [25]. PCR assays with the primers 5′CS and 3′-CS combined with forward and reverse primers for *bla*_VIM_ were carried out to determine the location of the *bla*_VIM_ gene within the class 1 integron [26]. A PCR assay with a forward primer for IS*1999* and reverse for OXA-48 was carried out to identify the presence of an insertion sequence upstream of *bla*_OXA-48_ [27]. The I*SEcp* insertion sequence was detected through PCR mapping with a forward primer for IS*Ecp* and universal reverse primer for *bla*_CTX-M_ genes (MA 3) [28]. Amplified products were detected through gel electrophoresis using 1% agarose gel and stained with ethidium bromide.

### 2.5. Conjugation and Plasmid Characterization

Conjugation experiments were performed by using *E. coli* J65AziR, resistant to sodium azide, and *E. coli* A15R-, resistant to rifampicin, as recipients [29]. Briefly, overnight cultures of donor and recipient strains were mixed 1:2 in brain–heart infusion broth and placed on MacConkey agar plates supplemented with either ertapenem (0.5 mg/L) or cefotaxime (2 mg/L) to inhibit the growth of the recipient strain and sodium azide (100 mg/L) or rifampicin (256 mg/L) to suppress the growth of the donor cells. Serial dilutions (10^−2^, 10^−4^ and 10^−6^) of the donor and recipient strain and mating mixture were prepared in saline and seeded on the previously mentioned plates in order to determine conjugation frequency. The plasmid profiling was achieved through PCR-based replicon typing (PBRT). Inc-type replicons were detected as previously reported [30,31].

### 2.6. Genotyping

Eight *K. pneumoniae* and two *E. cloacae isolates* were subjected to genotyping through multilocus sequence typing (MLST) in order to determine the sequence types (ST) according to the Pasteur website (multilocus sequence typing—MLST) databases and software for *K. pneumoniae*. Seven housekeeping genes were amplified, and the PCR products were detected using agarose gel electrophoresis, purified and sequenced using the Eurofin service. The obtained sequences were deposited into the above-mentioned website in order to obtain the ST (available online: https://bigsdb.pasteur.fr/klebsiella/ (accessed on 1 March 2023).

## 3. Results

### 3.1. Patients and Isolates

All but one patients were hospitalized at the moment of sample taking. Of the ten patients, eight were male and two were female. The age range was 1 to 77 and the median was 56 years. Nine patients presented clinical symptoms (urinary tract infection-UTI, bloodstream infection, wound infection) and one was colonized; all had severe underlying diseases. The urinary tract was the dominant source of the tested isolates. Three patients had chronic kidney failure, two solid organ transplantation and one vesicureteral reflux, UTI, a severe form of COVID-19, non-Hodgkin lymphoma and cerebrovascular insult, respectively. None of the patients had travelled internationally within the three months prior to the acquisition of the resistant strain. There were three patients hospitalized in the medical ICU, two in nephrology and one in hematology, urology, surgical intensive care unit (ICU) and COVID-19 unit, respectively. One outpatient who was an organ transplant recipient was included. After release from the hospital, he brought samples to the Andrija Štampar Public Health Institute. Out of 27 double carbapenemase-positive isolates recorded in the hospital databases, 10 isolates with multiple carbapenemases were stored and available for analysis: eight *K. pneumoniae* and two *E. cloacae* complex. The MALDI-TOF scores of the two *E. cloacae* complex isolates were 2.14 and 2.16.

The rate of detection of double carbapenemases among total carbapenemase-producing Enterobacterales depended on the center. The Andrija Štampar Public Health Institute had two double carbapenemase producers in 2017 among 52 carbapenemase-producing Enterobacterales (CPE), which is 3.9%. The University Hospital Centre Split and University Hospital Centre Osijek did not have double carbapenemases before 2022. The rate of double carbapenemases among the total number of CPE in 2022 ranged from 0.4% (9/2114) in UHCZ, 0.5% in the Andrija Štampar Public Health Institute and 0.8% (2/78) in UHCS to 1.09 (1/91) in UHCO. UHCZ, as the largest hospital center in Croatia and which provided the majority of isolates, had a drop in double carbapenemases during the pandemic, with rates of 1.33% in 2020 (2/15), 0.95%, (10/1047) in 2021 and 0.4% (9/2114) in 2022. Unfortunately, the isolates from 2020 and 2021 were not stored and are not available for further analysis. The year 2014 is not in the hospital database and, thus, there are no data on the total number of CPE. After one isolate recorded in 2014, UHCZ did not have isolates with double carbapenemases until 2020. The susceptibility patterns of the isolates from 2020 to 2021 that were not stored, obtained in the routine diagnostic procedures, are shown in Table S1 (Supplementary Materials). There was a drastic rise in the number of double carbapenemase producers between 2020 and 2021. Screening of the hospital internet database revealed that the combination of KPC with NDM was dominant in the pandemic period (2020–2021), identified in 7 out of 12 isolates (58%), followed by OXA-48+NDM, found in 4 isolates (33%). The rarest combination was OXA-48+VIM, with only one strain being positive. A shift to the domination of OXA-48+NDM was recorded after the pandemic. There was also a shift in the species harboring double carbapenemases. In the pandemic period, they were mainly harbored by *Citrobacter freundii,* whereas later, after the pandemic, *K. pneumoniae* prevailed.

### 3.2. Antimicrobial Susceptibility Testing and Phenotypic Tests for Detection of ESBLs, Plasmid-Mediated AmpC- β-Lactamases and Carbapenemases

The isolates demonstrated uniform resistance to amoxicillin, amoxicillin/clavulanate, piperacillin/tazobactam, cefuroxime, ceftazidime, cefotaxime, ceftriaxone, cefoxitin and ertapenem. High resistance rates were observed for cefepime and meropenem—90% (9/10)—amikacin, ciprofloxacin and trimethoprim/sulfamethoxazole—80% (8/10)—and gentamicin and imipenem—70% (7/10)—as shown in Table 1. Aztreonam demonstrated moderate activity, with four strains being susceptible. There was a varying level of susceptibility/resistance to carbapenems, with MICs ranging from 1 to >128 µg/mL. Colistin presented good activity, with only three isolates (30%) being resistant (Table 1). Among the novel β-lactam/inhibitor combinations, ceftazidime/avibactam exhibited moderate activity, with 50% of isolates susceptible. Cefiderocol was the most active compound, with MICs of all isolates in the susceptible range. All isolates demonstrated resistance to imipenem/cilastatin/relebactam and all but one to ceftolozane/tazobactam. Four isolates exhibited the MDR phenotype, whereas six were allocated to the XDR phenotype. DDST demonstrated negative results in all isolates, but for the combined disk test with clavulanate, it tested positive in two isolates, as shown in Table 1. The inhibitor-based test with PBA did not yield any positive results. The modified Hodge test demonstrated activity against meropenem and ertapenem in all isolates. CIM tested positive in all but one strain (Table 1). The inhibitor-based EDTA test yielded positive result in six isolates, suggesting the production of MBL. OKNV detected three combinations of carbapenemases: OXA-48+NDM (five isolates), OXA-48+VIM (three isolates) and OXA-48+KPC (two isolates).

### 3.3. Detection of Resistance Genes with Inter-Array Chip Technique

A great variety of different β-lactamases was identified among isolates. OXA-48 was confirmed in all tested isolates, with five isolates coharboring NDM, two KPC and one VIM. *bla*_CTX-M-15_ genes were identified in two isolates, with positive results in the combined disk test with clavulanic acid and three phenotypically negative for an ESBL. *bla*_TEM_ genes were detected in six isolates (Table 2). All *K. pneumoniae* isolates harbored intrinsic *bla*_SHV_ genes. Seven isolates tested positive for *bla*_OXA-1_ and one for *bla*_OXA-2_ genes encoding narrow-spectrum OXA-1 and OXA-2 β-lactamases with predominant penicillinase activity, whereas OXA-9 was found in two isolates, as shown in Table 2. Genes encoding OXA-18 and OXA-60 each occurred in only one strain. The *bla*_CMY_ gene was detected in one *K. pneumoniae* phenotypically negative for Amp-C β-lactamases. Plasmid-mediated *aac*(6) genes responsible for aminoglycoside resistance were found in four *K. pneumoniae* and two *E. cloacae* complex isolates, whereas *aad*A1 tested positive in one *K. pneumoniae* isolate. The genes encoding panaminoglycoside resistance *grm* and *rmt* were identified in eight and in three isolates, respectively. Plasmid-encoded fluoroquinolone resistance determinants, the *qnr*B and *qnr*S genes, were detected in three and one isolates, respectively (Table 2). The *Sul*1 gene encoding sulphonamide resistance was carried by seven isolates, with one being susceptible to trimethoprim/sulfamethoxazole. Six isolates expressed *dfr*A genes responsible for trimethoprim resistance, with five variants identified: *dfra*A5, *dfra*A7, *dfra*A14, *dfr*A17 and *dfr*A19 (Table 2). Genes for efflux pumps (*oqxa* and *oqxb*) were found in seven isolates (all *K. pneumoniae*). One *E. cloacae* complex isolate encoded *mcr*8 and *mcr*9 genes in spite of being susceptible to colistin. All but one MBL strains harbored class 1 integron.

**Table 1:** Minimum inhibitory concentrations, susceptibility category, phenotypic tests for β-lactamase detection and β-lactamase content of *Enterobacterales* isolates.

Protocol Number	Center	Date of Isolation	Specimen	OKNV	ESBL	Hodge/CIM	CXM	CAZ	CTX	CRO	FEP	IMI	MEM	GM	CIP	COL	C/T	CZA	IMR
KPN1	UHCZ Medical ICU	27 May 2022	urine	OXA-48+NDM	-	+/+	>128	>128	>128	>128	128	128	128	32	64	1	>256	0,25	>32
KPN 2	UHCZ Medical ICU	21 November 2022	blood culture	OXA-48+NDM	-	+/-	>128	>128	>128	>128	32	8	32	128	64	0.5	>256 >256	>256	>32
KPN3	UHCZ Surgical ICU	11 May 2022	urine	OXA-48+VIM	+	+/+	>128	64	>128	>128	16	1	1	1	>128	0.5	0.5	<0.016	>32
KPN4	UHCZ COVID-19 unit	16 May 2022	throat swab	OXA-48+VIM	+	+/+	>128	>128	>128	>128	128	1	8	16	>128	0.5	>256	0.016	>32
KPN 5	UHCS nephrology	8 April 2022	urine	OXA-48+KPC	-	+/+	>128	>128	>128	>128	128	128	128	0.5	1	64	6	0.12	4
KPN6	UHCS nephrology	8 August 2022	urine	OXA-48+KPC	-	+/+	>128	>128	>128	>128	>128	>128	>128	64	>128	64	>256	0.12	>32
KPN7	UHCZ Haematology	5 October 2022	wound swab	OXA-48+NDM	-	+/+	>128	>128	>128	>128	>128	>128	>128	32	>128	32	>256	32	>32
KPN8	Štampar outpatient	8 September 2017	urine	OXA-48+NDM	-	+/+	>128	>128	>128	>128	32	4	4	0.5	0.5	1	>256	32	8
ECL1	UHCOS Medical ICU	21 September 2022	urine	OXA-48+NDM	-	+/+	>128	>128	>128	>128	64	1	4	>128	32	0.25	>256	>256	4
ECL2	UHCZ urology	17 January 2014	urine	OXA-48+VIM	-	+/+	>128	>128	>128	>128	32	16	16	32	16	0.25	>256	>256	4

Abbreviations: KPN—*Klebsiella pneumoniae*; ECL—*Enterobacter cloacae* complex*;* OKNV—RESIST-4 O.K.N.V immunochromatographic assay; ESBL—inhibitor based test with clavulanic acid for detection of extended-spectrum β-lactamases; CIM—carbapenem inactivation method; CXM—cefuroxime; CAZ—ceftazidime; CTX—cefotaxime; CRO—ceftriaxone; FEP—cefepime; IMI—imipenem; MEM—meropenem; GM—gentamicin; CIP—ciprofloxacin; COL—colistin, C/T—ceftolozane–tazobactam; CZA—ceftazidime–avibactam; IMR—imipenem–-cilastatin–relebactam. UHCZ—University Hospital Centre Zagreb, UHCS—University Hospital Centre Split, UHCOS—University Hospital Centre Osijek, Štampar—Andrija Štampar Public Health Institute. The abbreviations of the antibiotics are in concordance with those provided in the EUCAST protocol. MIC resistance breakpoints according to CLSI are as follows: cefuroxime ≥32 µg/mL; cefepime and ceftazidime ≥16 µg/mL; gentamicin and ceftazidime/avibactam ≥8 µg/mL; cefotaxime, ceftriaxone, imipenem and meropenem, ≥4 µg/m; ciprofloxacin ≥1 µg/mL and colistin, imipenem/cilastatin/relebactam and ceftolozane/tazobactam >2 µg/mL (EUCAST).

### 3.4. PCR and Sequencing of bla Genes

PCR and sequencing identified the *bla*_CTX-M-15_ allelic variant, belonging to cluster 1, in six isolates (one negative in inter-array analysis), *bla*_TEM-1_ genes in six and *bla*_SHV-1_ in all eight *K. pneumoniae* isolates. IS*Ecp* was found upstream of *bla*_CTX-M-15_ genes in four out of six positive isolates. *bla*_VIM-1_ genes were amplified in three isolates, including two negative isolates in the inter-array test. All OXA-48 positive isolates generated the *bla*_OXA-48_ allelic variant. Both the *bla*_KPC_ genes encoded the KPC-2 allelic variant. *bla*_NDM_ genes were confirmed through PCR in all five isolates as being positive in the inter-array test, but the sequence was too short to determine the variant. IS*1999* was found upstream of the *bla*_OXA-48_ gene in four isolates (two *K. pneumonia* and two *E. cloacae* complex). Class 1 integron was amplified in seven out of eight MBL-containing organisms, including the following isolates: KPN1, KPN2, KPN3, KPN4, KPN 7, ECL1 and ECL2. Only one MBL-positive strain KPN8 tested negative for class 1 integron.

### 3.5. Conjugation and Characterization of Plasmids

Neither cefotaxime nor ertapenem resistance were transferable from any of the two isolates phenotypically positive for an ESBL, and all ten positive for carbapenemases, respectively, with either of the two recipient strains. A wide variety of different plasmid Inc types were found among the isolates. The IncL plasmid was identified in six isolates, whereas IncA/C and IncX were found in three and one isolates harboring the NDM-containing combination, respectively. Two isolates positive for KPC had the IncFIIs plasmid. IncW and IncHI1 were each found only in one isolate.

### 3.6. Genotyping by Multilocus Sequence Typing

*K. pneumoniae* isolates exhibited genetic diversity and belonged to five different STs: KPC containing isolate belonged to ST 1789, whereas those with VIM were assigned to ST437 and ST39. The NDM-including combinations were ST 101 and 147. Three isolates could not be assigned to any ST using the conventional method, but the nearest match is shown in Table 2. Two *E. cloacae* isolates belonged to ST114 and ST754.

## 4. Discussion

There was an increase in CPE isolates during the COVID 19 pandemic in UHCZ, the largest hospital center in Croatia, with 15 isolates in 2020, 1047 in 2021 and 2114 in 2022, but the rate of double carbapenemases against the total number of CPE decreased from 1.3% in 2020 to 0.4% in 2022. The double carbapenemase producers were not recorded in UHCZ before 2020, except for one strain identified in 2014, which could be due to the increased antibiotic consumption during the COVID-19 pandemic. The hospital centers outside Zagreb did not have such isolates before 2022. The dominant combination was OXA-48+NDM, which is in concordance with a multicenter study recently carried out in European countries. All combinations contained OXA-48, which is the most widespread type of carbapenemase in Croatia in the last decade, significantly outnumbering the KPC and VIM variants [7,8,9,32]. KPC was the additional carbapenemase detected in the isolates from Split, whereas those from Zagreb and Osijek contained MBL and OXA-48. The increased usage of antibiotics during the pandemic exerted a selection pressure that favored the accumulation of resistance genes by Enterobacterales. In 2020, there was a very low number of carbapenemase-producing organisms recorded, and only two isolates were positive for double carbapenemases. This is probably due to lockdown leading to reduced hospitalization and invasive procedures. Comparison of the isolates analyzed in the present study with those recorded in the hospital database revealed a switch from the predominance of KPC with NDM in the pandemic years (2020–2021) to the domination of OXA-48 with NDM in the postpandemic period (2022). The species harboring double carbapenemases also exhibited a switch from the dominance of *C. freundii* in the pandemic years to *K. pneumoniae* postpandemic. The isolates harboring multiple carbapenemases analyzed in this study belonged into two species: *K. pneumoniae* and *E. cloacae* complex. These species are known to be good vectors for plasmids. There are a lot of strains positive for carbapenemases in our ICUs, as described in the previous studies [3,4,5,6,7,8,9]. It is possible that cross infections occurred between patients in the same hospital ward. Since the majority of the patients had strains with a single carbapenemase, for example, *K. pneumoniae* with OXA-48 and *E. cloacae* with NDM, it is possible that the conjugation occurred in vivo in the gut of the patients, enabling the acquisition of additional carbapenemase genes due to exchange of plasmids. This was not proven in this study. Whole-genome sequencing could probably explain the origin of these isolates, but this was beyond this study.

The isolates contained various resistance genes to β-lactam and non-β-lactam antibiotics. There was a discrepancy between the inter-array chip and OKNV results in two isolates (KPN3 and KPN4). OKNV identified two carbapenemases (VIM and OXA-48) in two *K. pneumoniae* isolates in which the chip identified only OXA-48. However, PCR and sequencing confirmed the OKNV results. These two isolates exhibited low MICs of imipenem and meropenem (mostly in the susceptible range) and a negative EDTA test, which is consistent with OXA-48 positivity, leading to the conclusion that *bla*_VIM_ genes were not expressed. Moreover, the inter-array test failed to detect CTX-M β-lactamase in one isolate (KPN3) that was phenotypically positive for an ESBL and PCR-positive for *bla*_CTX-M_ genes. *bla*_CTX-M_ genes were preceded by the IS*Ecp* insertion sequence, responsible for the mobilization of the gene. However, the *bla*_CTX-M_ genes were not transferable to *E. coli* recipient strains from any of the tested isolates, regardless of the presence of the insertion sequence. *bla*_carb_ genes were not transferable either. This could be due to the large size of the L plasmid carrying the OXA-48 encoding gene or inappropriate recipient strains.

The inhibitor-based tests exhibited low sensitivity (33%) in detecting an ESBL in the isolates with a combination of carbapenemases. The high rate of false negativity could be due to the masking effect of carbapenemase on an ESBL. Moreover, the inhibitor-based tests with PBA failed to detect CMY plasmid-mediated AmpC β-lactamase in one *K. pneumoniae* strain. The CIM and Hodge test exhibited high sensitivity, but the EDTA inhibitor-based test failed to recognize two VIM-producing organisms.

The predominance of the IncL plasmid is in line with OXA-48 positivity in all isolates.

MLST revealed the diversity of STs, which is in line with the fact that all cases were sporadic and recovered from different geographic regions. Six isolates from UHCZ were all from the medical ward, but without any link between the cases. Previous studies identified VIM-1 in combination with KPC-2 in an outbreak reported in Germany in 2011 involving *K. pneumoniae* [33]. A recent report from Turkey described a combination of OXA-48 and KPC and KPC and NDM [34]. A multicenter study carried out in Europe last year proved the international spread of *K. pneumoniae* clone-harboring OXA-48 and NDM-1, which was the most frequent combination in our study as well [35,36]. In a European study, international travel, particularly to Serbia and Turkey, was the most important source of the isolates, and Germany was the most-affected country, likely due to high migration rates. However, in our study, none of the patients had stayed abroad prior to the acquisition of the resistant isolate, indicating that our strains developed de novo under the selection pressure of antibiotics. The first country reporting *K. pneumoniae* with multiple carbapenemases was Greece in 2009 [37,38]. The extensive usage of antibiotics in the COVID-19 pandemic increased the rate of such difficult-to-treat strains. Similarly, as in our study, ST-1789 was associated with KPC-2 in a previous study conducted in northern Italy [39]. Conversely, ST 437 linked to the combination of OXA-48 and VIM was found in a previous study in NDM-positive *K. pneumoniae* in China [40]. Combinations of OXA-48 with KPC were confined to the Mediterranean region of Croatia, while in Zagreb and Slavonia, combinations with MBLs were identified. None of the STs reported in this study were present in Croatia before.

Recipients of solid organ transplants and patients receiving cytotoxic chemotherapy are routinely and extensively given broad-spectrum antibiotics as prophylaxis. Antibiotics exert a selective pressure favoring the horizontal spread of resistance determinants. Moreover, the use of medical devices enables pathogens to colonize patients and develop biofilms. Similarly, patients with vesicoureteral reflux often have relapsing UTIs and are given multiple antibiotic treatments.

## 5. Conclusions

To our knowledge, this is the first report of the co-occurrence of two carbapenemases in the same isolate from Croatia. This study demonstrated the ability of *K. pneumoniae* to acquire various resistance determinants over time. The presence of MBLs compromises the use of ceftazidime/avibactam and ceftolozane/tazobactam. Only cefiderocol exhibited promising activity. The OKNV results showed correlation with PCR, but the inter-array chip failed to detect some of the β-lactamases identified through other methods. *mcr* genes encoding plasmid-mediated, transferable resistance to colistin were reported for the first time in Croatia in the strain susceptible to colistin, indicating non-functional genes. There are many gaps in our understanding of the development of antibiotic resistance in *K. pneumoniae* and its excellent capacity to accumulate resistance determinants. The species has special biological significance, likely associated with specific antibiotic selection pressure, pathogenic fitness, gene mobility and other attributes. This study provided insight into the abundance of resistance genes and ongoing evolution of resistance in *K. pneumoniae.* Since the majority of isolates exhibited the XDR phenotype, close surveillance of *K. pneumoniae* should be considered for continuous monitoring of new resistance phenotypes in this ultimate superbug.

## Figures and Tables

**Table 2:** Inter-array analysis of *K. pneumoniae* and *E. cloacae* complex isolates with multiple carbapenemases. ND—not determined, ST—sequence type.

Protocol Number	β-Lactamase Content	Aminoglycoside Resistance Genes	Fluoroquinolone Resistance Genes	Sulphonamide and Trimethoprim Resistance Genes	Efflux Pump Genes	Colistin Resistance Genes	ST
KPN1	SHV, OXA-1, I*SEcp*CTX-M-15, OXA-48, NDM	*aac(6)Ib*		*Sul 1, dfrA14*	*oqxa, oqxb*		ND (closest 3750 or 5859)
KPN 2	SHV, OXA-1, OXA-48, IS*Ecp*CTX-M-15, NDM	*aac(6)Ib, grm*		*sul1, dfrA5*	*oqxa, oqxb*		ST-101
KPN3	TEM, SHV, OXA-48	*grm, aph*	*qnrB*	*sul1. sul2, dfrA7, dfraA14, dfrA17*	*oqxa, oqxb*		ST-39
KPN4	TEM, SHV, OXA-1, OXA-18, OXA-60, I*SEcp*CTX-M-15, OXA-48	*aac(6), grm*			*oqxa, oqxb*		ST-437
KPN 5	OXA-48, KPC				*oqxa, oqxb*		ND (closest 101 or 647)
KPN6	TEM, SHV, OXA-9, OXA-48, KPC,	*armA, grm, rmtA*			*oqxa, oqxb*		ST-1789
KPN7	TEM, SHV, OXA-1 IS*Ec*pCTX-M-15, OXA-9, CMY, OXA-48, NDM	*aac(6)Ib, aadA1, armA, aphA, grm*	*qnrS*	*Sul1, sul2, dfrA5*	*oqxa, oqxb*		ST-147
KPN8	SHV, OXA-1, OXA-48, NDM	*grm*		*Sul1*			ND (closest 1 or 47)
ECL1	OXA-1, OXA-2, OXA-48, NDM	*aac(6)Ib, aac′6II, aac(6)IIc, grm*	*qnrB*	*Sul1, dfrA5, dfrA19*		*mcr 8, * *mcr 9*	ST114
ECL2	TEM, OXA-1, OXA-48, VIM, I*SEcp*CTX-M-15	*aac(6)Ib, aadA1, grm*	*qnrB*	*Sul1, sul2, dfrA5, dfrA14*			ST754

## Data Availability

The data presented in this study are available on request from the corresponding author.

## Supplementary Materials

The following supporting information can be downloaded at: https://www.mdpi.com/article/10.3390/pathogens12050677/s1; Table S1: Antibiotic susceptibility of isolates with double carbapenemases originating from 2020-2021. The data are downloaded from the hospital data base and are based on disk-diffusion testing. MICs are given in parenthesis only for imipenem, meropenem and colistin. OKNV testing was done for the purpose of routine laboratory diagnostic.
