# Supplementary material for: Emergence and Spread of Enterobacterales with Multiple Carbapenemases after COVID-19 Pandemic
Source: Pathogens. 2023 May 3;12(5):677. doi: 10.3390/pathogens12050677 (PMC10223518; doi:10.3390/pathogens12050677)
Supplement: Supplementary file 1 [file pathogens-12-00677-s001.zip › pathogens-2275986-supplementary.docx]

**Table S1**. Antibiotic susceptibility of isolates with double carbapenemases originating from 2020–2021. The data are downloaded from the hospital data base and are based on disk-diffusion testing. MICs are given in parenthesis only for imipenem, meropenem and colistin. OKNV testing was done for the purpose of routine laboratory diagnostic.

Abbreviations: CF-*Citrobacter freundii*; KPN-*Klebsiella pneumoniae*; ECL-*Enterobacter cloacae;* OKNV- RESIST-4 O.K.N.V immunochromatographic assay; AMC-amoxycillin/clavulanic acid; TZP-piperacillin/tazobactam; CXM-cefuroxime; CAZ-ceftazidime; CTX-cefotaxime; CRO- ceftriaxone; FEP-cefepime; IMI-imipenem; MEM-meropenem; GM-gentamicin; CIP-ciprofloxacin; COL-colistin, C/T-ceftolozane-tazobactam; CZA-ceftazidime-avibactam; IMR-imipenem-cilastatin-relebactam. UHCZ-University Hospital Centre Zagreb,

|  | **Protocol number** | **Center** | **Date** | **OKNV** | **AMC** | **TZP** | **CXM** | **CAZ** | **CTX** | **CRO** | **FEP** | **IMI** | **MEM** | **GM** | **CIP** | **COL** | **C/T** | **CZA** | **IMR** |
| --- | --- | --- | --- | --- | --- | --- | --- | --- | --- | --- | --- | --- | --- | --- | --- | --- | --- | --- | --- |
| 1 | CF 159975 | UHCZ | 29/06/2020 | KPC+NDM | ND | ND | ND | ND | ND | ND | ND | ND | ND | ND | ND | ND | ND | ND | ND |
| 2 | KP 236505 | UHCZ | 21/09/20 | OXA-48+NDM | ND | ND | ND | ND | ND | ND | ND | ND | ND | ND | ND | ND | ND | ND | ND |
| 3 | KP 180209 | UHCZ | 23/06/2021 | KPC+NDM | R | R | R | R | R | R | R | R  (128) | R  (128) | R | R | S  (0,25) | ND | R | ND |
| 4 | CF 126252 | UHCZ | 29/06/2021 | KPC+NDM | R | R | R | R | R | R | R | R  (128) | R  (128) | R | R | S  (0,5) | ND | R | ND |
| 5 | CF  185527 | UHCZ | 01/07/2021 | KPC+NDM | R | R | R | R | R | R | R | R  (128) | R  (128) | R | R | S  (0,5) | ND | R | ND |
| 6 | CF  189385 | UHCZ | 01/07/2021 | KPC+NDM | R | R | R | R | R | R | R | R  (128) | R  (128) | R | R | S  (0,5) | ND | R | ND |
| 7 | CF  223500 | UHCZ | 03/08/2021 | KPC+NDM | R | R | R | R | R | R | R | R  (64) | R  (128) | R | R | S  (0,5) | ND | R | ND |
| 8 | CF  224890 | UHCZ | 04/08/2021 | KPC+NDM | R | R | R | R | R | R | R | R  (64) | R  (16) | R | R | S  (2) | ND | R | ND |
| 9 | CF  249502 | UHCZ | 31/08/2021 | KPC+NDM | R | R | R | R | R | R | R | R  (64) | R  (128) | R | R | S  (0,5) | ND | R | ND |
| 10 | ECL  229186 | UHCZ | 09/08/2021 | OXA-48+NDM | R | R | R | R | R | R | R | R  (16) | R  (128) | R | R | S  (0,5) | ND | R | ND |
| 11 | KPN  331729 | UHCZ | 08/11/2021 | OXA-48+NDM | R | R | R | R | R | R | R | R  (128) | R  (128) | R | R | S  (0,5) | ND | R | ND |
| 12 | KPN  332903 | UHCZ | 08/11/2021 | OXA-48+NDM | R | R | R | R | R | R | R | R  (64) | R  (128) | R | R | S  (0,5) | ND | R | ND |
